# An unexpected effect of TNF-α on F508del-CFTR maturation and function

**DOI:** 10.12688/f1000research.6683.2

**Published:** 2015-09-02

**Authors:** Sara Bitam, Iwona Pranke, Monika Hollenhorst, Nathalie Servel, Christelle Moquereau, Danielle Tondelier, Aurélie Hatton, Valérie Urbach, Isabelle Sermet-Gaudelus, Alexandre Hinzpeter, Aleksander Edelman

**Affiliations:** 1 Inserm U1151, Team 2 - CNRS UMR 8253, Faculté de Médecine Paris Descartes, Institut Necker Enfants Malades, Paris, 75993, France; 2 INSERM U955, Team 5, Université Paris Est Créteil, Champs-sur-Marne, 77420, France

**Keywords:** Cystic fibrosis, epithelium, F508del-CFTR, inflammation, tumor necrosis factor-alpha, chloride channel, CFTR, correctors

## Abstract

Cystic fibrosis (CF) is a multifactorial disease caused by mutations in the cystic fibrosis transmembrane conductance regulator gene ( *CFTR),* which encodes a cAMP-dependent Cl ^-^ channel. The most frequent mutation, F508del, leads to the synthesis of a prematurely degraded, otherwise partially functional protein. CFTR is expressed in many epithelia, with major consequences in the airways of patients with CF, characterized by both fluid transport abnormalities and persistent inflammatory responses. The relationship between the acute phase of inflammation and the expression of wild type (WT) CFTR or F508del-CFTR is poorly understood. The aim of the present study was to investigate this effect. The results show that 10 min exposure to TNF-alpha (0.5-50ng/ml) of F508del-CFTR-transfected HeLa cells and human bronchial cells expressing F508del-CFTR in primary culture (HBE) leads to the maturation of F508del-CFTR and induces CFTR chloride currents. The enhanced CFTR expression and function upon TNFα is sustained, in HBE cells, for at least 24 h. The underlying mechanism of action involves a protein kinase C (PKC) signaling pathway, and occurs through insertion of vesicles containing F508del-CFTR to the plasma membrane, with TNFα behaving as a corrector molecule. In conclusion, a novel and unexpected action of TNFα has been discovered and points to the importance of systematic studies on the roles of inflammatory mediators in the maturation of abnormally folded proteins in general and in the context of CF in particular.

## Introduction

Cystic fibrosis (CF) is a genetic disease attributable to mutations in the cystic fibrosis transmembrane conductance regulator gene ( *CFTR). CFTR*’s main function is encoding a cAMP-dependent Cl ^-^ channel. The most frequent mutation, F508del, leads to the synthesis of a prematurely degraded, otherwise partially functional protein. *CFTR* is expressed in many epithelia, but the most important consequences of mutated *CFTR* are in the airways, ascribed to both abnormal fluid transportation and excessive inflammatory responses. These abnormalities lead to the bacterial colonization of the lung, causing lung obstruction and resulting ultimately in respiratory insufficiency and death. The primary origin of this inflammatory scenario has been controversial for a long time. Dealing with this question in 2009, we wrote “…many authors consider it secondary to recurrent infections and airway colonization by opportunistic pathogens” ^1^. Today, a growing body of evidence indicates that inflammation and infection in CF can be dissociated, and that a basal inflammatory status preexists pathogen infections ^2^. Pezzulo and colleagues ^2^, studying the relationship between ion transport in trachea and inflammation/infection, showed that inflammation results from bacterial infection and is independent from CFTR function. Nevertheless, reports from 2015 show that inflammation precedes infection in the CF ferret model ^3^.

Different studies have established a direct link between ion transport regulation and inflammation ^1, 4^. However, there is still insufficient knowledge about how the mediators of inflammation modulate CFTR expression, and consequently, if they modulate ion transport. Furthermore, most of the previous works in this area were performed in cell models over-expressing wild-type (WT) CFTR ^1, 5– 8^. These studies showed that cytokines could either reduce ^6^, or increase ^1^ CFTR expression and function depending on the cell type and treatment duration. In Calu-3 cells derived from a pulmonary adenocarcinoma, treatment of cells for more than 24h (corresponding to chronic inflammation conditions) with a pro-inflammatory cytokine (TNFα) activated *CFTR* gene expression at the transcriptional level ^7^, whereas the same treatment reduced CFTR expression in a colon adenocarcinoma-derived cell line (T84) ^6^. The impact of cytokine treatment on epithelial ion permeability was addressed by another study, showing the involvement of complex transduction signaling pathways concerning different mitogen-activated protein (MAP) kinases ^8^.

Even less information exists about the effects of cytokines on CFTR during the acute phase of inflammation. We have previously observed that short-term (10min) treatment of Calu-3 cells by TNFα induces CFTR-dependent eicosanoid production, and CFTR-independent IL-1β secretion ^1^. Additionally, these observations may be extended to the context of F508del/F508del patients, as we have reported that residual activity of CFTR in the nasal epithelium exists in patients with a mild phenotype, suggesting that inflammatory status may be correlated with residual CFTR function ^9^. We hypothesize now that cytokines could affect the expression and function of mutated CFTR during the acute phase of inflammation, being in part responsible for this residual activity. The aim of this study was to evaluate the effects of acute and chronic stimulation by TNFα or IL-1β on F508del-CFTR in two cell types: HeLa cells stably expressing F508del-CFTR, and primary human bronchial epithelial cells (HBE) derived from F508del homozygous patients.

## Materials and methods

### Reagents and chemicals

Human recombinant cell culture grade TNF was purchased from Jena Bioscience GmbH (Jena, Germany); Brefeldin A (BFA; B7651), forskolin (from *Coleus forskohlii*; F6886), amiloride hydrochloride hydrate (A7410-5G); inh-172 (C2992-5MG) and genistein (G6649-5MG) were purchased from Sigma-Aldrich (St Quentin Falavier, France); a protein kinase C inhibitor ^10^, GF109203X, was purchased from Selleckchem (USA, S7208). Anti-CFTR antibodies (abs): MM13-4 mouse monoclonal ab against N-terminus of CFTR, (Millipore, France, 05-581); 24-1 mouse monoclonal ab against C-terminus of CFTR (R&D Systems, MAB, 25031). Anti-tubulin abs: rabbit polyclonal anti-tubulin ab (Ab4074) and anti-NaKATPase mouse monoclonal ab (Ab7671) were from Abcam (Paris, France). For western blot analysis, the secondary polyclonal goat anti-mouse abs were IRDye ^®^ 800CW (Li-Cor, Bad Homburg, Germany, 926-32210), anti-keratin 8 mouse monoclonal abs (Progen, Biotechnik GmbH, Heidelberg, Germany, 61038), and anti-NHERF1 (rabbit polyclonal Santa-Cruz Biotechnology, B2107). For immunocytochemistry, anti–Zona occludens 1 (ZO-1) rabbit polyclonal abs were purchased from Santa Cruz Biotechnology, sc-10804; secondary anti-mouse (A-11001) and anti-rabbit (A-24923) Alexa-fluor (488 and 594) IgGs were from Life technologies (Fontenay s/Bois, France). For proximity ligation, the secondary abs were provided by OLINK in the kit (DUO92004 for Duolink ^®^ in Situ PLA ^®^ Probe anti-mouse MINUS, and DUO92002 for Duolink ^®^ in Situ PLA ^®^ Probe anti-rabbit PLUS, Bioscience, Uppsala, Sweden). *In situ* kits for proximity ligation assay were purchased from OLINK. Human primary bronchial epithelial (HBE) cells in air-liquid interface are cultivated on microporous filters purchased from Corning Incorporated (Transwell ^®^ polyester membrane cell culture inserts, 6.5mm diameter, New York, USA). IL1β was obtained from ENZO life sciences (ALX-520-001-C010, Villeurbanne, France).

## Cell culture

### TNFα treatments

***HeLa cell culture.*** HeLa cells stably transfected with the wild type CFTR plasmid construct and the mutated F508del-CFTR were kindly provided by Pascale Fanen (INSERM U955, Créteil, France). Cells were cultured in Dulbecco’s modified Eagle medium, supplemented with 10% FCS, 2mM glutamine, 100g/ml streptomycin, 100 units/ml penicillin and 0.25mg/ml Zeocin (all reagents were from Invitrogen) in an incubator at 37°C and 5% CO _2_.

***Primary Human bronchial epithelial (HBE) cell culture.*** Primary HBE cells were isolated from bronchial explants of CF and non-CF patients after lung transplantation, with consent approved by Comité de protection des personnes Ile de France II, 2010-05-03 A3. Cells were isolated from bronchial tissue by enzyme digestion and were cultured in differentiation medium (DMEM/F12, supplemented with 2% UltroserG) on type I collagen-coated filters. Briefly, bronchial explants were washed twice with washing medium (Eagle’s minimum essential medium + antibiotics (2.5µg/ml amphotericin B, 150µg/ml piperacillin plus tazobactam, 25µg/ml ciprofloxacin) + dithioteitol (DTT) + DNAse) and at least twice with (Eagle’s minimum essential medium (MEM) containing only antibiotics (as above) to remove DTT. Bronchial explants were then incubated for 24h in a MEM medium containing antibiotics (150µg/ml piperacillin plus tazobactam, 25µg/ml ciprofloxacin), Amphotericin B and protease, at 4°C with constant rotation (1500rpm for 5min). Next day, 15% fetal calf serum was added to neutralize proteases, and bronchia with medium were placed on a Petri dish. Epithelial cells were scraped with a curved scalpel from the inner surface of bronchia, centrifuged (1500rpm, 7min, 4°C) and resuspended in trypsin (incubation 10min). After that, differentiation medium (MEM) with serum (FCS) was added and cells were re-centrifuged. Cells were resuspended in an appropriate volume of FCS medium (DMEM/F12, 5% FCS, non-essential amino acids, appropriate antibiotics depending on the patient’s clinical status) and counted. The cells were plated with 10 ^6^ cells/cm ^2^ to cover apical surface of each filter coated as described above. UG2% medium (DMEM/F12, supplemented with 2% Ultroser G, appropriate antibiotics (amphotericin B, tazocillin, ciprofloxacin, concentration as above) was added to the basal side of filters. The next day, apical medium (FCS) was aspirated and cells were gently washed (to remove cells other than epithelial) with PBS-antibiotics. Starting from the second day of culture, the basal medium was changed daily. Basal medium which passed to the apical compartment was removed daily. Cells were cultured at an air-liquid interface for at least 21 days and were differentiated to form polarized epithelium, after 2 weeks of growth ^11^. Cell differentiation was verified with immunofluorescent staining of markers: α-tubulin for ciliated cells, Zona-occludens (ZO-1) for tight junctions, keratin 8 (K8) for simple epithelia marker, mucin 5AC (MUC5AC) for goblet cells and CFTR. The transepithelial resistance (R _T_) of cultures was measured and short circuit current (Isc) experiments were performed on cultures with at least 800Ω/cm ^2^.

TNFα was added to the culture medium for periods of time indicated in the Results section without fetal calf serum except for patch-clamp experiments.

***Protein sample preparation for CFTR immunoblotting.*** Sample preparation protocols are described in detail elsewhere ^12, 13^. Briefly, cells were washed on ice twice with phosphate-buffered saline (PBS) solution containing 0.1mM CaCl _2_ and 1mM MgCl _2_. PBS (Mg ^2+^- and Ca ^2+^-free) was added to scraped cells. A first centrifugation was done at 1500g at 4°C. Cells were resuspended in a hypotonic solution containing 10mM KCl, 10mM TRIS at pH7.4, 1.5mM MgCl _2_ and homogenized with a mini Potter-Elvehjem tissue grinder. Cells were centrifuged at 15000g at 4°C for 15min. The resulting supernatant was re-centrifuged for 1h at 100000g at 4°C. The pellet was resuspended in a hypotonic solution. The total amount of protein was quantified by Lowry-Folin assay ^14^.

***Western blot analysis.*** Western blot analyses were performed as described elsewhere with slight modifications ^15^. Briefly, equal amounts of proteins/lane were electrophoresed on an 8% SDS-PAGE and electrotransferred onto nitrocellulose membranes over 2h at 4°C in Tris-glycine buffer (Biorad) at 200 mA. Next, nitrocellulose membranes were incubated in PBS + 0.1% tween20 (Sigma, 9005-64-5) containing 5% milk (Regilait Bio, Supermarket Simply, Paris) saturation solution for 1h. Proteins were immunoblotted for 2h with the MM-13-4 ab (1/1000). After extensive washing, the nitrocellulose membranes were incubated with anti-tubulin (1/5000) or anti-NaKATPase (1/5000) abs. Next, the last washes (three times for 30min in washing buffer: PBS plus 0.1% Tween20) were done. CFTR, tubulin or NaK-ATPase were detected using Odyssey detection system (Li-Cor, Bad Homburg, Germany). The relative protein expression was assessed using the ImageJ 1.47v software ( http://imagej.nih.gov/ij/index.html).

***Whole-cell patch-clamp recordings.*** The technique for patch-clamp recordings in the whole-cell configuration has been described elsewhere ^16, 17^. Stably transfected cells were plated in 35-mm glass bottom plates that were mounted on the stage of an inverted microscope. Patch experiments were performed at room temperature with an Axopatch 200A amplifier controlled by a computer *via* a digidata 1440 interface (Axon Instruments, USA). Pipettes were pulled from hard glass (Kimax 51) using a Sutter micropipette puller, and the tips were fire-polished. Current recordings were performed using the nystatin-perforated patch-clamp configuration ^16^. The nystatin stock solution (50 mg/ml) was prepared daily in DMSO. The stock solution was diluted (1:250) with the internal solution, which was sonicated for 1min. The internal solution contained the following (in mM): 131 NaCl, 2 MgCl _2_ and 10 Hepes, pH 7.3 adjusted with NaOH. The bath solution contained (in mM): 150 NaCl, 1 CaCl _2_, 1 MgCl _2_, 35 sucrose and 10 Hepes-Na ^+^, pH 7.3, adjusted with NaOH.

Currents were recorded by application of regular pulses of -60 mV for 1s, with a holding potential of 0 mV and an interval of 3 s.

To establish the I-V curves, regular voltage pulses were interrupted by a series of 9 voltage jumps (1-s duration each) toward membrane potentials between -100 and +80 mV. CFTR Cl ^-^ currents, I _CFTR_, were activated using 400 µM 8-(4-chlorophenylthio)-cAMP sodium salt (CPT-cAMP) and 100 µM 3-isobutyl-1-methylxanthine (IBMX).

When maximal stimulation was reached, cells were bathed with 5 to 50ng/ml of TNFα in the presence of CPT-cAMP and IBMX TNFα solution, and steady-state was achieved after 7 to 10 minutes.

Then 5 µM of the CFTR inhibitor, CFTR _inh_172, was added to the CPT-cAMP containing perfusion solution (solution +/- TNFα). I _CFTR_, defined CFTR currents as a difference in current amplitude recorded during maximum stimulation with solution +/- TNFα and maximum inhibition with CFTR _inh_172. Data were analyzed using the Student’s t-test (Origin Pro 9.1 software, RITME, France); results were considered to be statistically significant if the *p* value was less than 0.05 (for non-parametric tests, the Mann-Whitney U test was used).

***Short-circuit current experiments.*** For short-circuit current measurements, primary human bronchial epithelial cells (HBE) were grown on permeable filters (0.33-cm ^2^ surface area) at an air-liquid interface for differentiation and then inserts were mounted in Ussing chambers (Physiologic Instruments, San Diego, CA). For all measurements, a Cl ^-^ gradient was applied by differential composition of basal and apical Ringer solutions. The basal Ringer solution contains: 145mM NaCl, 3.3mM K _2_HPO _4_, 10mM HEPES, 10mM D-Glucose, 1.2mM MgCl _2_, and 1.2mM CaCl _2_; and apical solution contains: 145mM Na-Gluconate, 3.3mM K _2_HPO _4_, 10mM HEPES, 10mM D-Glucose, 1.2mM MgCl _2_, 1.2mM CaCl _2_. Cells were washed for a 30-min stabilization period in Ringer solutions and aerated with 95% O _2_/5% CO _2_ at 37°C. Transepithelial resistance (R _T_) was measured by applying a 15mV pulse and calculating R _T_ by Ohm’s Law. Isc was measured with an EVC4000 Precision V/I Clamp (World Precision Instruments) and registered using a PowerLab 4/30 workstation (AD Instruments, Castle Hill, Australia). During continuous recording of Isc (in voltage-clamp mode) various inhibitors and activators were added. After stabilization of baseline Isc, amiloride (100µM) was added to the apical side of inserts to inhibit the apical epithelial sodium channel (ENaC). Then Forskolin (10µM) and IBMX (100µM) were added to apical and basolateral compartments, followed by Genistein (50µM) and then CFTR inhibitor Inh-172, added apically at a 5µM concentration.

***Immunocytochemistry.*** HeLa cells and polarized epithelial monolayers of HBE cells were fixed with ice-cold acetone for 5min, then rinsed twice with PBS. Permeabilization was done with PBS containing 0.1% Triton X100 for 15min (PBS-T). Cells were then incubated in blocking solution (3% BSA in PBS-T) for 20min. CFTR immuno-detection by confocal microscopy (see below) was performed with p.24-1 antibody diluted 1/300 (for HeLa cells) or 1/100 (for primary HBE cells) in blocking solution, during overnight incubation at 4°C. Accompanying K8 or ZO-1 staining were done simultaneously. Following this, cells were washed four times for 5min each in PBS-T 0.1% and blocked in 10% goat serum (in PBS-T). Goat secondary IgGs conjugated to Alexa 488 and 594 were added for 30min at 1/1000 dilution in 10% goat serum. After a final four washes for 5min each, Vectashield mounting medium containing DAPI (Vector Laboratories, H-1200) was used to mount cells on microscope slides.

***Confocal microscopy.*** Cells were visualized and images captured using Leica TCS SP5 AOBS confocal microscope (Heidelberg, Germany), equipped with 63x/1.4 oil differential interference contrast λ blue PL APO objective. Typically we performed multiple optical xy sections over the cell culture to reconstitute using the ImageJ software v.147, and the 3D reconstitution of polarized epithelia of HBE cells was performed with 3D Viewer plugin in ImageJ.

***DNA proximity ligation assay.*** Cells were grown on round microscopy cover slips and fixed with ice-cold acetone for 5min, then rinsed twice with PBS. In the first step of the proximity ligation assay (PLA) procedure, cells were incubated in bovine serum albumin solution (blocking solution provided by O-link) for 30min at 37°C and then with either two primary anti-keratin-8 mouse monoclonal abs, or mouse monoclonal anti-NHERF1 ab and rabbit polyclonal anti-CFTR ab for 1h at 37°C. After three washes with PBS-T 0.1%, cells were incubated for 1h at 37°C with the PLA probes (secondary abs provided in the kit) specific to mouse and rabbit IgGs, coupled to the oligonucleotides. Cells were then washed three times and incubated with a mixture of ligase and oligonucleotide-connectors (sequences homologous to the oligonucleotides conjugated to PLA probes). Connectors hybridize with PLA probes only when the distance is <40 nm and form a circle which is enzymatically ligated. Following this, polymerase and nucleotides coupled to fluorochromes were added for amplification of circular oligonucleotides as a template, using the PLA probe sequences as primers. Each step of this protocol is separated by washing with PBS-0.1% tween20 solution to remove non-specific interactions. At the end of this procedure, cells were mounted on microscope slides with Vectashield mounting medium containing DAPI and signal was detected as fluorescent orange spots. PLA results are quantitative and presented as number of spots per cell.

***Statistical analysis.*** Experiments were repeated at least three times and analyzed using the unpaired non-parametric Student’s t-test (Mann-Whitney U test) using Graphpad Prism 5 or Origin (see in patch-clamp section).

## Results

### TNFα increases cell surface expression of F508del-CFTR in HeLa cells

**Figure 1.**
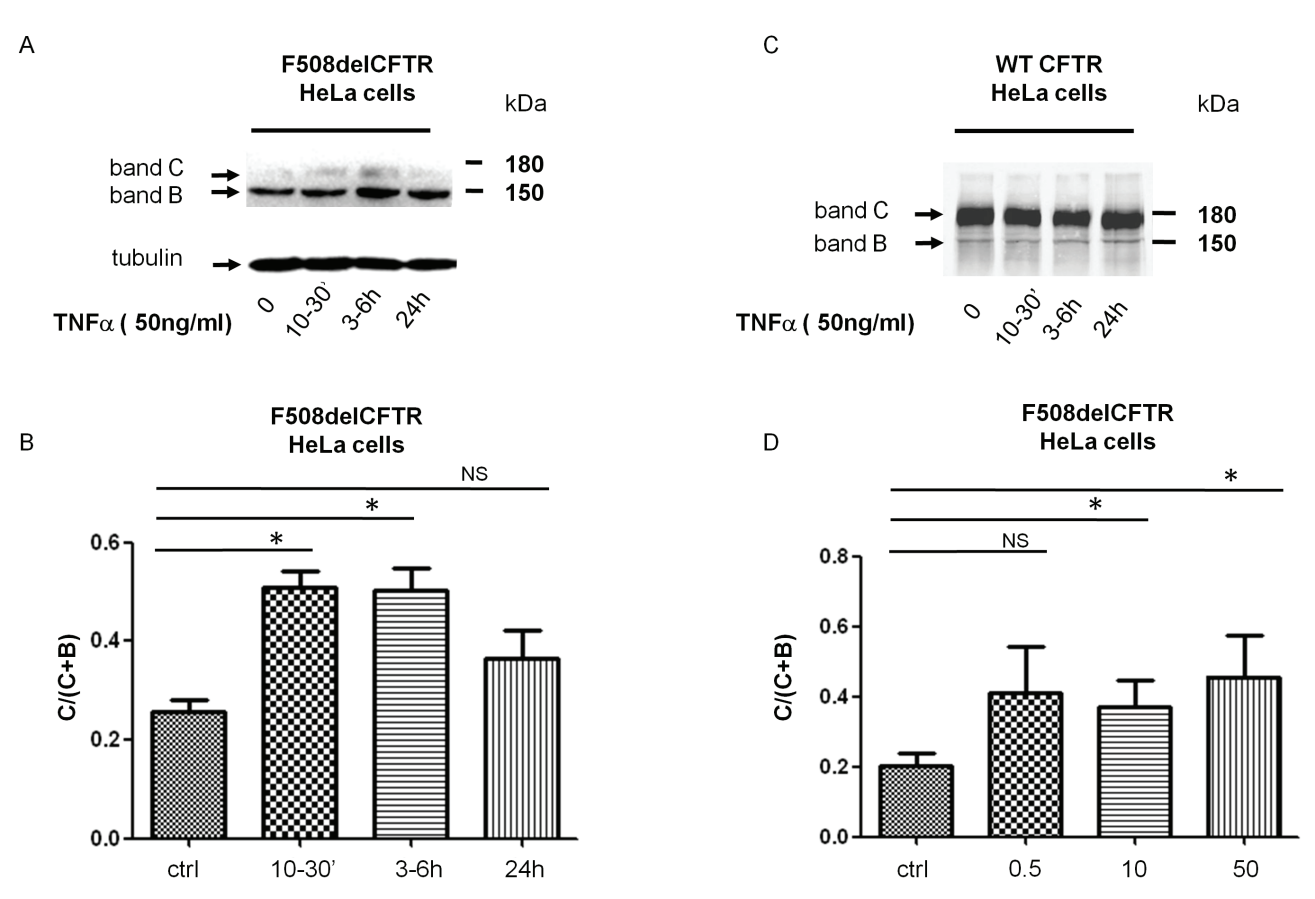
Immunoblot analysis of F508del-CFTR and WT-CFTR expression in HeLa cells. * indicates significant results. **A**. F508del-CFTR expression after stimulation of cells with 50ng/ml TNFα for 10min, 3–6h and 24h. Band C refers to fully glycosylated F508del-CFTR, band B refers to core glycosylated F508del-CFTR. **B**. Relative quantification of C/B+C indicating changes in maturation of F508del-CFTR after stimulation of cells with 50ng/ml TNFα for 10min, 3-6 and 24 h, p=0,0046, p=0,0234, p=0,69 (NS) for 10mins, 3–6h and 24 h, respectively. **C**. WT-CFTR expression after stimulation of cells with 50ng/ml TNFα for 10min, 3–6h and 24h. **D**. Concentration dependence of expression and the changes in maturation of F508del-CFTR in response to treatment of cells with 0.5, 10 and 50ng/ml TNFα, p=0.12 (NS), p=0.01, p=0.03, respectively.

We first investigated the effect of acute TNFα treatment on HeLa cells stably transfected with F508del-CFTR as a function of time. A representative immunoblot is shown in ( Figure 1A) and the relative quantification in Figure 1B. The analysis of microsomal proteins showed that the fully glycosylated mature CFTR (band C) could be detected after a 10–30min treatment with 50ng/ml of TNF *α*. The effect persisted for 3–6h and decreased after 24h of treatment, suggesting that TNFα might have a very rapid correcting effect on misfolded F508del-CFTR. The same treatment performed on HeLa stably expressing WT-CFTR was without effect ( Figure 1C). We then tested if the effect of TNFα was concentration-dependent. Figure 1D shows the quantification of immunoblot analysis of proteins derived from F508del-CFTR HeLa cells treated with different concentrations of TNFα, ranging from to 50ng/ml for 3–6 h. The relative quantification of mature (band C) *vs.* core-glycosylated F508del-CFTR (band B) showed a maximal effect at 0.5ng/ml TNFα, which did not increased significantly at higher concentrations ( Figure 1D).

**Figure 2.**
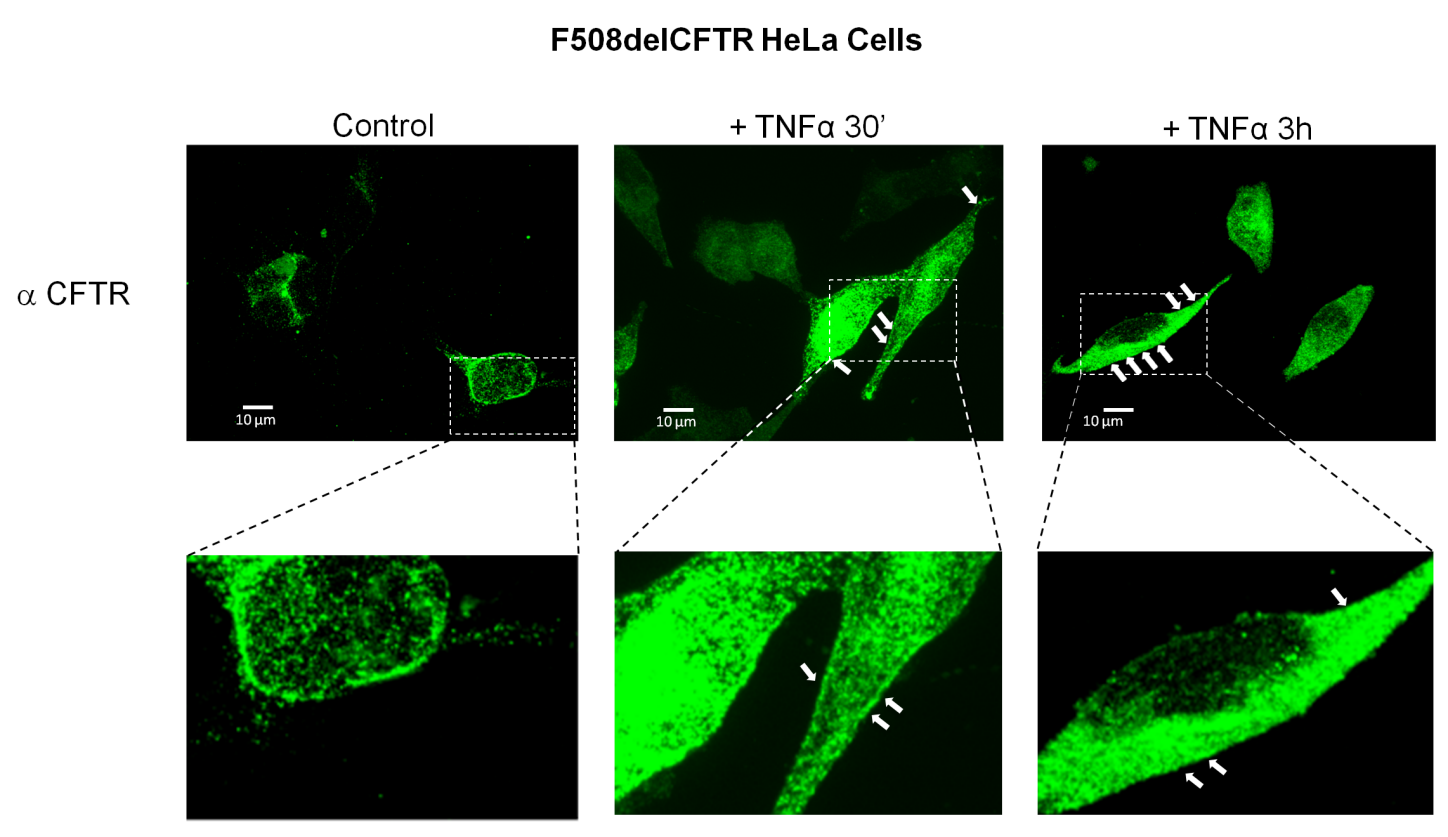
Localization of F508del-CFTR in HeLa cells. HeLa cells stably transfected with F508del-CFTR were subjected to CFTR immunodetection and analyzed by confocal microscopy (scale bar = 10 µm). Untreated cells (left panels). Cells treated with 50ng/ml TNFα for 30 min (middle panels). Cells treated with 50ng/ml TNFα for 3–6h (right panels). White arrows show a possible membrane localization of F508del-CFTR.

Immunoblot analysis data were supported by immunocytochemistry experiments. The treatment of F508del-CFTR expressing HeLa cells with 50ng/ml of TNFα for 3–6h resulted in a marked increase of CFTR staining suggesting an increase in F508del-CFTR expression and a possible relocalization of F508del-CFTR to the plasma membrane ( Figure 2, white arrows).

### TNFα restores function of F508del-CFTR in HeLa cells

**Figure 3.**
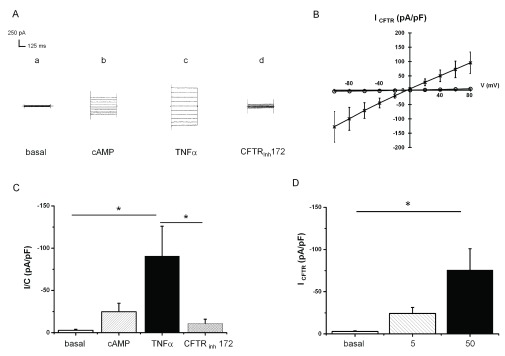
Effect of TNFα on whole-cell Cl ^-^ currents recorded in HeLa cells expressing F508del-CFTR by patch-clamp experiments. * indicates significant results. **A**. Representative current traces recorded by holding the membrane potential at 0 mV and by pulsing the voltages in the range -100 mV to +80 mV at 20 mV steps. Current traces recorded: at the basal level ( **a**); in the presence of CPT-cAMP/IBMX ( **b**); in the presence of 50 TNFα+CPT-cAMP/IBMX ( **c**); in the presence of 5 µM CFTR _Inh_172, 50ng/ml TNFα and 400 µM CPT-cAMP/IBMX ( **d**). **B**. Mean CFTR-related current amplitudes recorded at -60 mV and normalized to cell capacitance in the presence of CPT-cAMP/IBMX ( **O**); in the presence of 50 TNFα+CPT-cAMP/IBMX ( **X**). **C**. Mean current amplitudes recorded at -60 mV and normalized to cell capacitance (means + SEM, N=8): at the basal level; in the presence of CPT-cAMP/IBMX; in the presence of 50 TNFα+CPT-cAMP/IBMX; in the presence of 5 µM CFTR _Inh_172, 50ng/ml TNFα and 400 µM CPT-cAMP/IBMX. Wilcoxon signed rank test (paired samples): basal vs TNFα p=0.014, TNFα vs CFTR _inh_ 172 p=0.014. **D**. Dose-response of 0 to 50ng/ml TNFα after 10min: mean CFTR current amplitudes recorded at -60 mV and normalized to cell capacitance (means + SEM; ns for 5ng/ml N=4; p<0.05 for 50ng/ml n=8).

In the next series of experiments, we tested whether TNFα-induced delivery of F508del-CFTR to the plasma membrane was associated with CFTR-Cl ^-^ channel function. Using the nystatin-perforated patch-clamp configuration, we observed the activation of a cAMP-dependent Cl ^-^ current, which was sensitive to a CFTR inhibitor (inh _172_, 5μM) attesting to the presence of a CFTR current (I _CFTR_; Figure 3A, B and C) within 10–30min after addition of 5 or 50ng/ml TNFα to the solution ( Figure 3A, B and D). Non-treated control cells did not display I _CFTR_ ( Figure 3A). These experiments are in concordance with the biochemical data showing that acute TNFα translocates functional F508del-CFTR to the plasma membrane and therefore behaves like a corrector. Application of the same protocol to HeLa cells expressing WT-CFTR did not change the amplitude of I _CFTR_ (data not shown).

**Figure 4.**
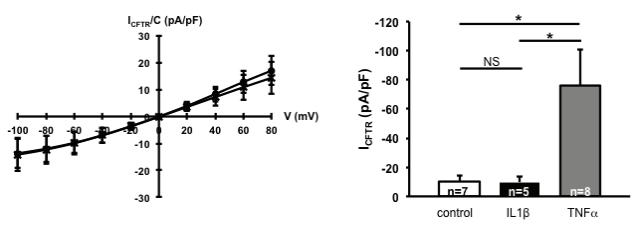
Effect of IL-1β on F508del-CFTR expressing HeLa cells. * indicates significant results. I _CFTR_ recorded in F508del-CFTR-expressing HeLa cells by patch clamp. Mean CFTR related current-voltage relationships recorded in the presence of 400 µM CPT-cAMP/100 µM IBMX ( **O**) and the presence of 10ng/ml IL1β + CPT-cAMP/IBMX ( **X**). The current was normalized to capacity (pA/pF), left panel. The normalized CFTR currents are depicted as mean + SEM, in absence (control, white column) or presence of 10ng/ml IL1β for 10min (IL1β, black column) or presence of 50ng/ml TNFα for 10min (TNFα, grey column). IL1β did not significantly increase I _CFTR_ compared to control (NS), but TNFα significantly increased I _CFTR_ compared to IL1β (p=0.03) and control (p=0.03; unpaired Student’s t-test), right panel.

To test whether other pro-inflammatory cytokines induce F508del-CFTR function, we tested the effects of different concentrations of IL-1β on F508del-CFTR-expressing HeLa cells. Treatment of cells with 10 ng IL-1β for 10–30min did not induce I _CFTR_ ( Figure 4).

### TNFα induces apical F508del-CFTR localization in human bronchial epithelial cells

**Figure 5.**
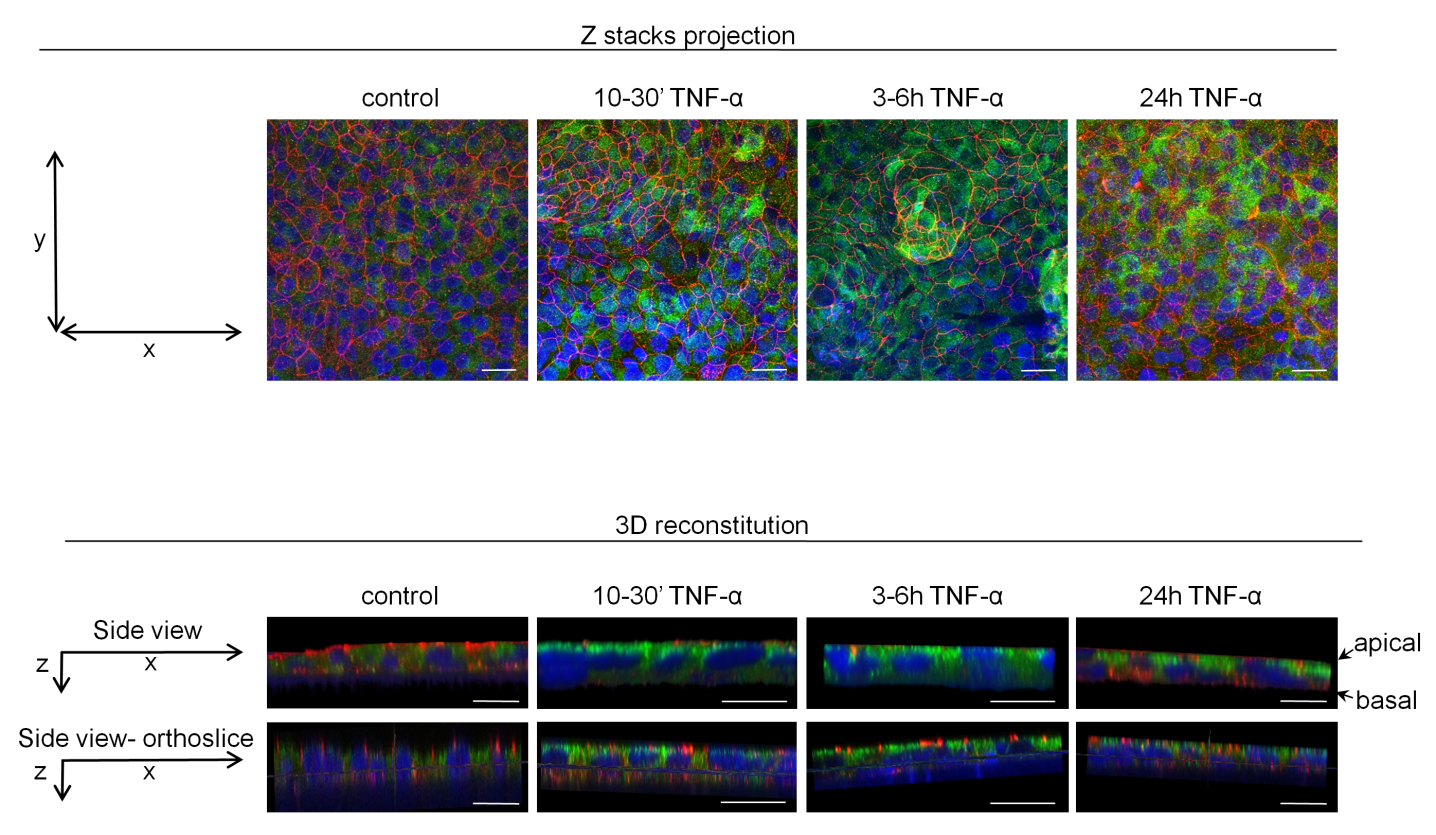
Increased apical localization of F508del-CFTR in primary HBE cell cultures upon treatment with TNFα. Differentiated primary HBE cell cultures grown at an air-liquid interface were incubated with 50ng/ml TNFα for 10–30min, 3–6h and 24h. CFTR immunodetection was performed with 24.1 anti-CFTR antibody and analyzed with confocal microscopy. Green staining represents CFTR (Alexa Fluor 488), red color staining represents ZO-1 protein of tight junctions (Alexa Fluor 594) and blue DAPI staining visualizes nuclei. Independent TNFα treatments and CFTR immunodetection were performed on HBE cells from three different F508del/F508del CF patients. Representative images of one experiment are demonstrated (Scale bars = 20µm).

To investigate whether the acute effects of TNFα on F508del-CFTR maturation may have physiological consequences, we performed experiments on primary human bronchial epithelial cells from CF patients homozygous for the F508del mutation, cultured at an air-liquid interface. Confocal microscopy analysis of F508del-CFTR distribution in reconstituted epithelium was performed. Figure 5 shows representative images obtained in HBE cell cultures from three different patients bearing F508del/F508del mutations. Green fluorescence, corresponding to the presence of CFTR protein, increased in cell preparations treated with TNFα (50ng/ml) compared to control, suggesting an increase in F508del-CFTR expression. Furthermore, in TNFα treated cells, F508del-CFTR appeared in the same plane as ZO-1, indicating its apical localization, in contrast to lighter and diffuse cytoplasmic staining in control conditions. The redistribution of F508del-CFTR to the apical side of epithelium occurred within 10min of TNFα 50ng/ml treatment and was sustained over 24h of treatment.

### TNFα restores functional CFTR in bronchial epithelial cells derived from F508del CF patients

**Figure 6.**
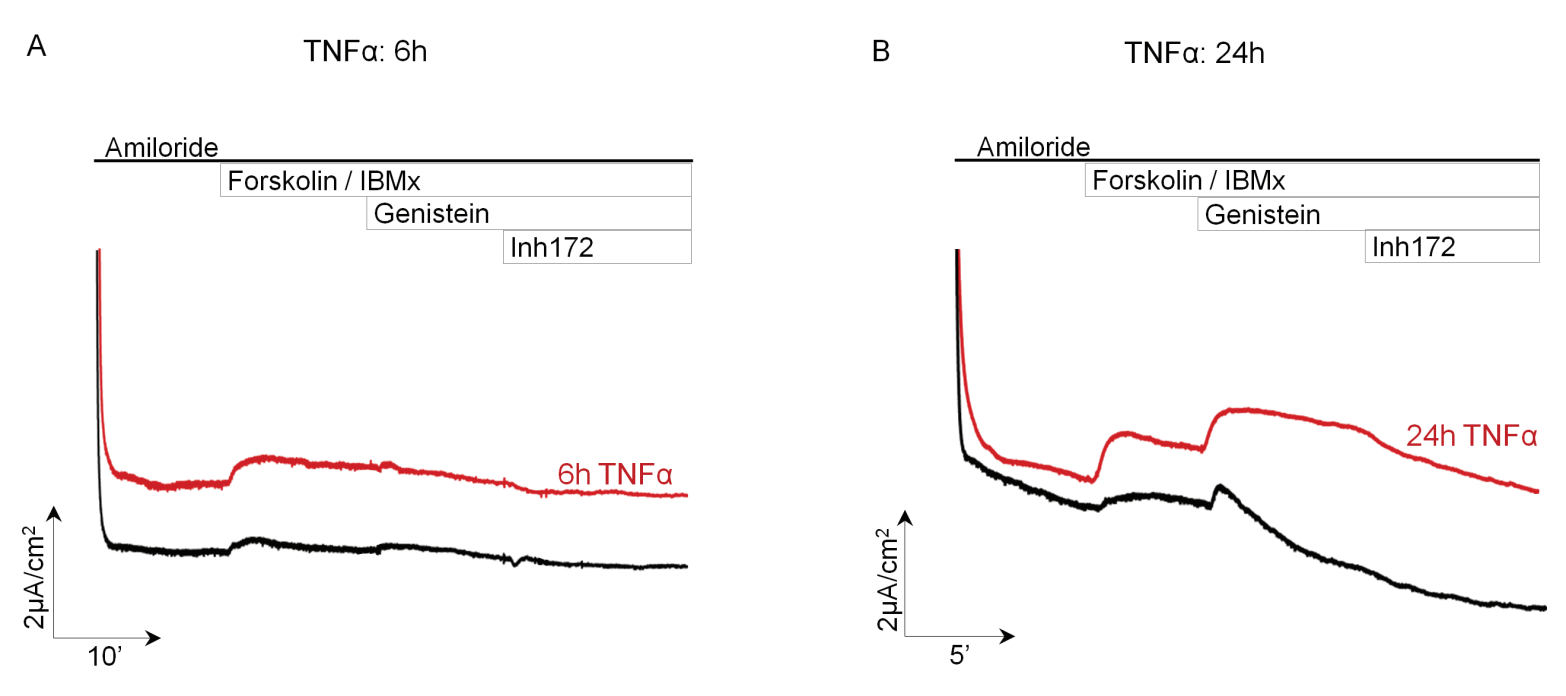
TNFα treatment of primary HBE cells increases CFTR-dependent chloride secretion. Short-circuit current experiments on air-liquid cultures of HBE cells from a F508del/F508del CF patient. Amiloride, an inhibitor of Na ^+^ current (ENaC), was present throughout the experiment. Forskolin (10µM) + IBMX (100µM) were added apically and basolaterally to increase the intracellular cAMP to activate the cAMP-dependent currents, followed by addition of genistein (50µM), a phytoestrogen known to potentiate I _CFTR_. The inhibitor of I _CFTR_, inh-172 (10µM), was added to the apical Ussing chamber to attest to the presence of I _CFTR_. **A**. Treatment of cells for 6h with 50ng/ml of TNFα induced cAMP-dependent, inh172 sensitive chloride current of higher magnitude than control non-treated cells. **B**. Treatment of cells for 24h with 50ng/ml of TNFα induced cAMP-dependent chloride current, potentiated by genistein and abolished by inh172 of higher magnitude than control cells. Black tracings represent Isc measurements in control untreated cell cultures whereas red tracings visualize Isc measurements in epithelia treated with TNFα.

We investigated the functional consequences of F508del-CFTR insertion in the plasma membrane upon TNFα exposure using Isc measurements. Representative Isc recordings are shown in Figure 6: TNFα treatments of CF HBE cells enhanced the cAMP-sensitive Isc, which is consistent with increased activity of CFTR, as compared to non-treated control cells. Increased responses to Forskolin ( Figure 6A and B) as well as potentiation by genistein or inhibition by Inh-172 ( Figure 6B) were observed. In these cells, the effect was still visible after 24h of incubation with TNFα ( Figure 6). Altogether, these experiments suggest that TNFα exerts a correcting effect during the acute and resolving phases of inflammation, by promoting rapid insertion of F508del-CFTR into the apical membrane of primary HBE cells derived from CF patients.

### TNFα effect on F508del-CFTR maturation: mechanism of action

To understand how TNFα enables the exit of F508del-CFTR from the ER, we investigated the role of several possible TNFα targets and signaling pathways, including ER to Golgi vesicular transport, keratin 8 and PKC-related signaling.

**Figure 7.**
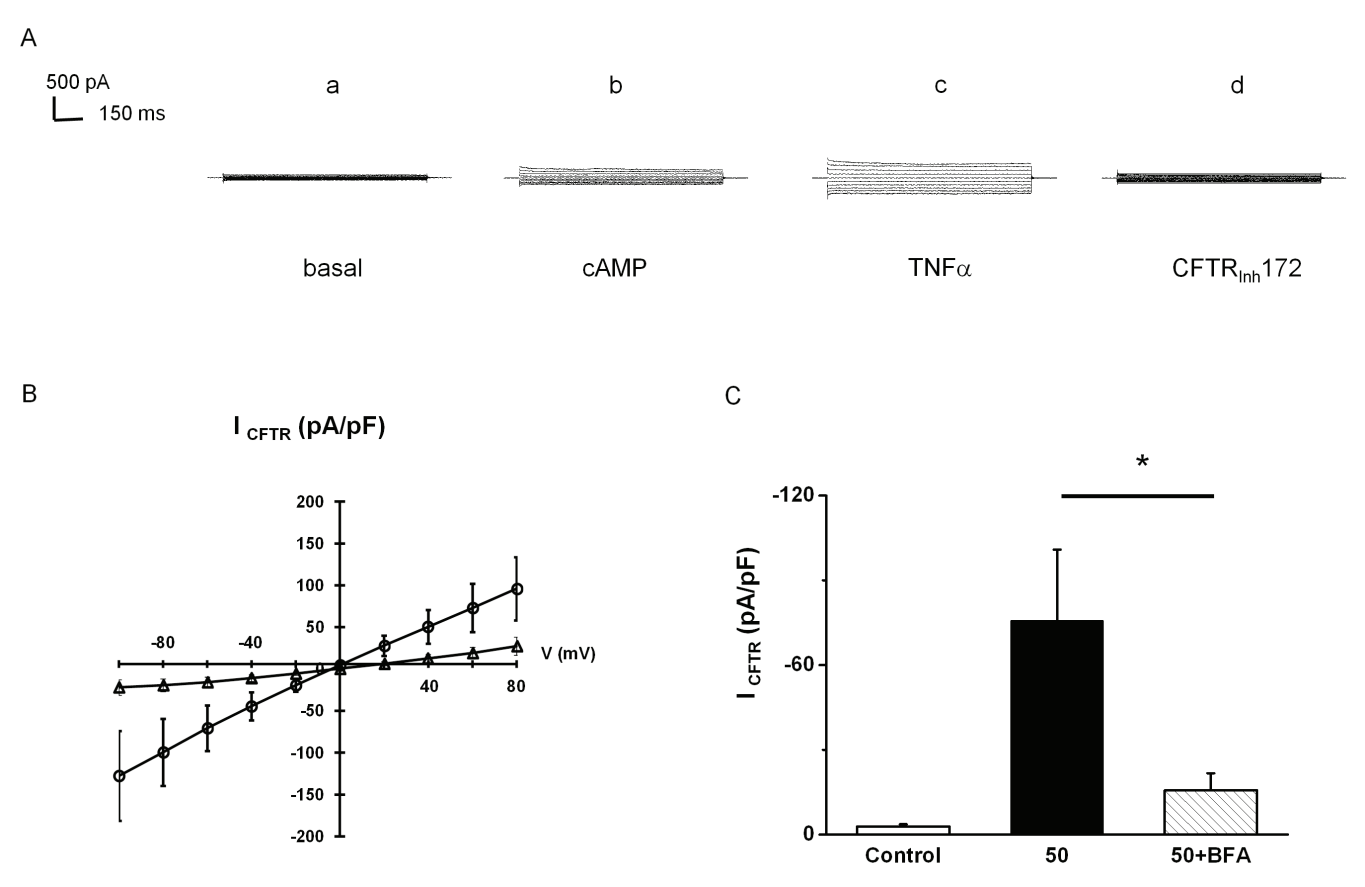
Stimulation of F508del-CFTR activity by 50 ng/ml TNFα is blocked by BFA. Whole cell Cl ^-^ currents recorded in HeLa cells expressing F508del-CFTR by patch-clamp experiments. * indicates significant results. **A**. Representative current traces recorded by holding the membrane potential at 0 mV and by pulsing the voltages in the range -100 mV to +80 mV at 20 mV steps for cells treated by 35 µM brefeldin A (BFA) for 2h. Current traces recorded: at the basal level ( **a**); in the presence of CPT-cAMP/IBMX ( **b**); in the presence of 50 TNFα+CPT-cAMP/IBMX ( **c**); in the presence of CFTR _Inh_172+50 TNFα+CPT-cAMP/IBMX ( **d**). **B**. Mean CFTR-related current-voltage relationships. Current densities normalized to cell capacitance (pA/pF) were measured in the presence of TNFα ( **O**); on cell treated for 2h by 35µM brefeldin A in the presence of TNFα (Δ). **C**. Mean CFTR current amplitudes recorded at -60 mV and normalized to cell capacitance in untreated cells (control; mean + SEM; n=8), in cells treated for 10 minutes by TNFα (50; mean + SEM, n=8) and in cells treated by 35 µM brefeldin A for 2h and for 10 minutes by TNFα (50+BFA; means + SEM, n=6). Statistics: unpaired Student’s t test between (50) and (50 + BFA), p=0.025.

In a first set of experiments, cells were pre-treated with BFA, (35µM for 3–4h), an inhibitor of protein transport from the ER to the Golgi apparatus ^18^. Pretreatment with BFA abolished I _CFTR_ induced by exposure of cells to 50ng/ml of TNFα ( Figure 7) indicating that vesicular trafficking is involved in TNFα action.

**Figure 8.**
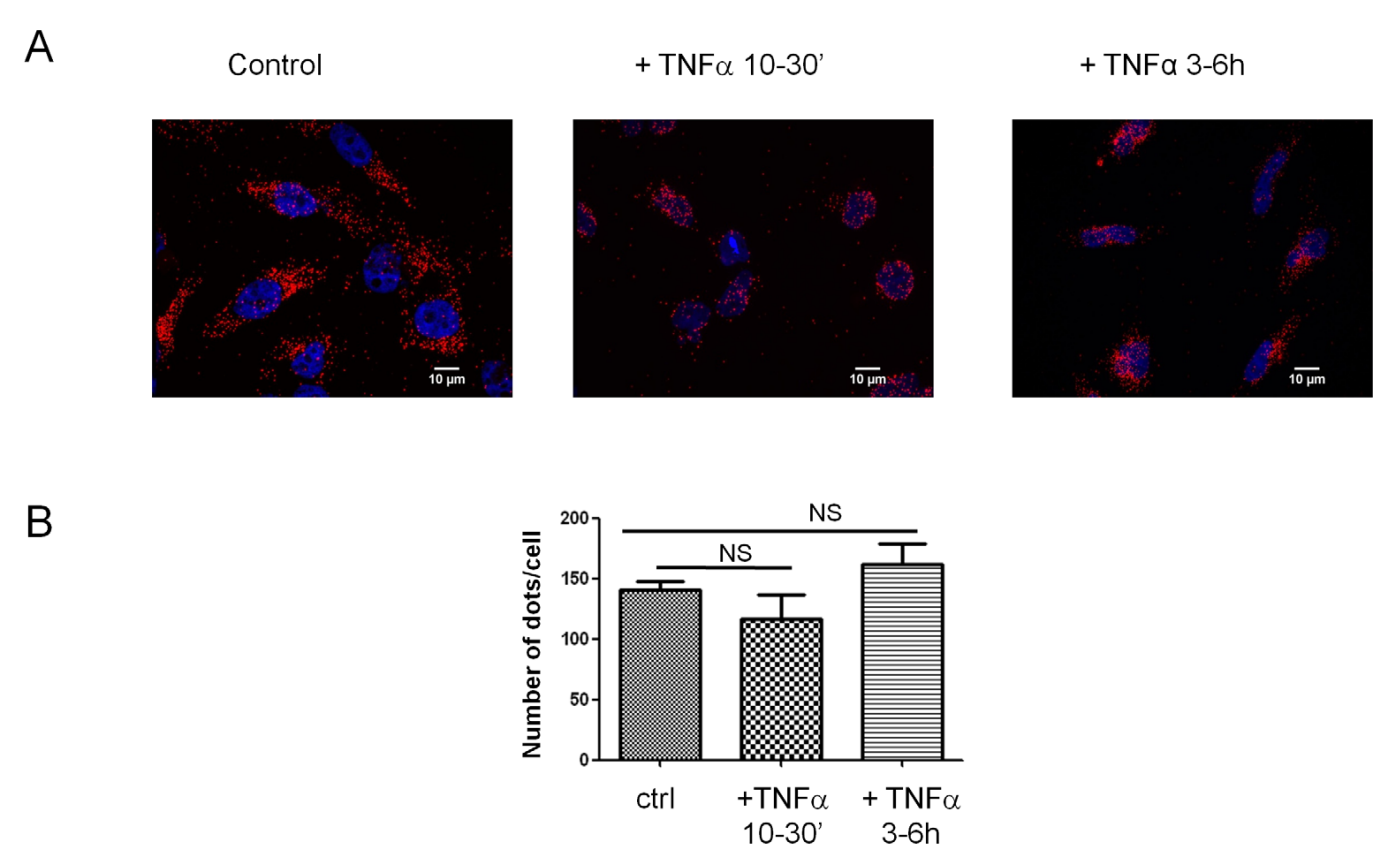
Keratin 8-F508del-CFTR proximity in HeLa cells. **A**. Differential interaction between K8 and CFTR in cells treated with TNFα (50ng/ml) for varied durations. DNA proximity ligation assay of K8 and CFTR in HeLa cells transfected with F508del-CFTR, scale bar = 10 µm. **B**. Number of dots corresponding for proteins pairs, Keratin 8 –F508del-CFTR, per cell. NS, not significant.

In a second set of experiments, the keratin-8 proximity to F508del-CFTR in HeLa cells +/- TNFα was evaluated. Using the PLA assay, we tested whether the number of K8-F508del-CFTR pairs that are closer than 40nm was changed by TNFα treatment. The results indicate that incubation with 50ng/ml TNFα for 30min or 3 h had no effect on the number K8-F508del-CFTR pairs ( Figure 8A and B) suggesting that the interaction between keratin 8 and F508del-CFTR was not a target of TNFα.

**Figure 9.**
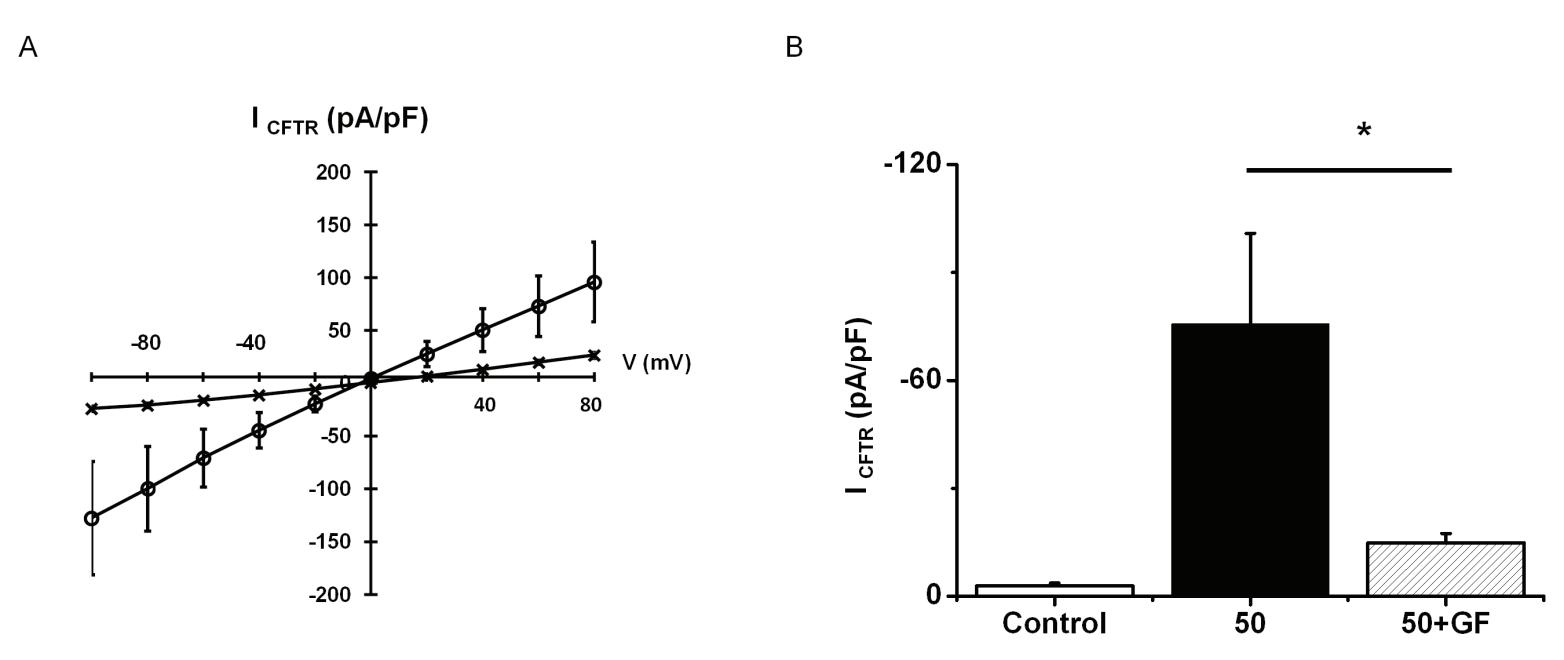
Stimulation of F508del-CFTR activity by TNFα is blocked by PKC inhibitor (GF109203X). Whole cell Cl ^-^ currents recorded in HeLa cells expressing F508del-CFTR by patch-clamp experiments. * indicates significant results. **A**. Mean CFTR-related current-voltage relationships. Current densities normalized to cell capacitance (pA/pF) were measured in the presence of 50 ng/ml TNFα ( **O**); on cells treated for 30 min by 5µM GF109203X in the presence of TNFα ( **X**). **B**. Mean CFTR current amplitudes recorded at -60 mV and normalized to cell capacitance in untreated cells (control; mean + SEM; n=8), in cells treated for 10 minutes by TNFα (50; means + SEM, n=8) and in cells treated by 5 µM GF109203X for 30min and for 10 minutes by TNFα (50+GF; means + SEM, n=6). Statistics: non-parametric unpaired Student’s t test between (50) and (50 + GF), p=0.047.

A third series of experiments was designed to investigate the possible role of protein kinase C (PKC) in TNFα action. TNFα has been reported to induce within 30min the insertion of the leptin B receptor into the plasma membrane in a PKC-dependent manner ^10^. To test if this is also the case for F508del-CFTR, cells were pre-treated for 2–4h with a PKC inhibitor, GF109203X. GF109203X prevented the TNFα-induced changes on I _CFTR_, suggesting that a PKC-dependent signaling pathway is involved in this process ( Figure 9A and B).

### Peripheral quality control by TNFα

**Supplementary Figure S1.**
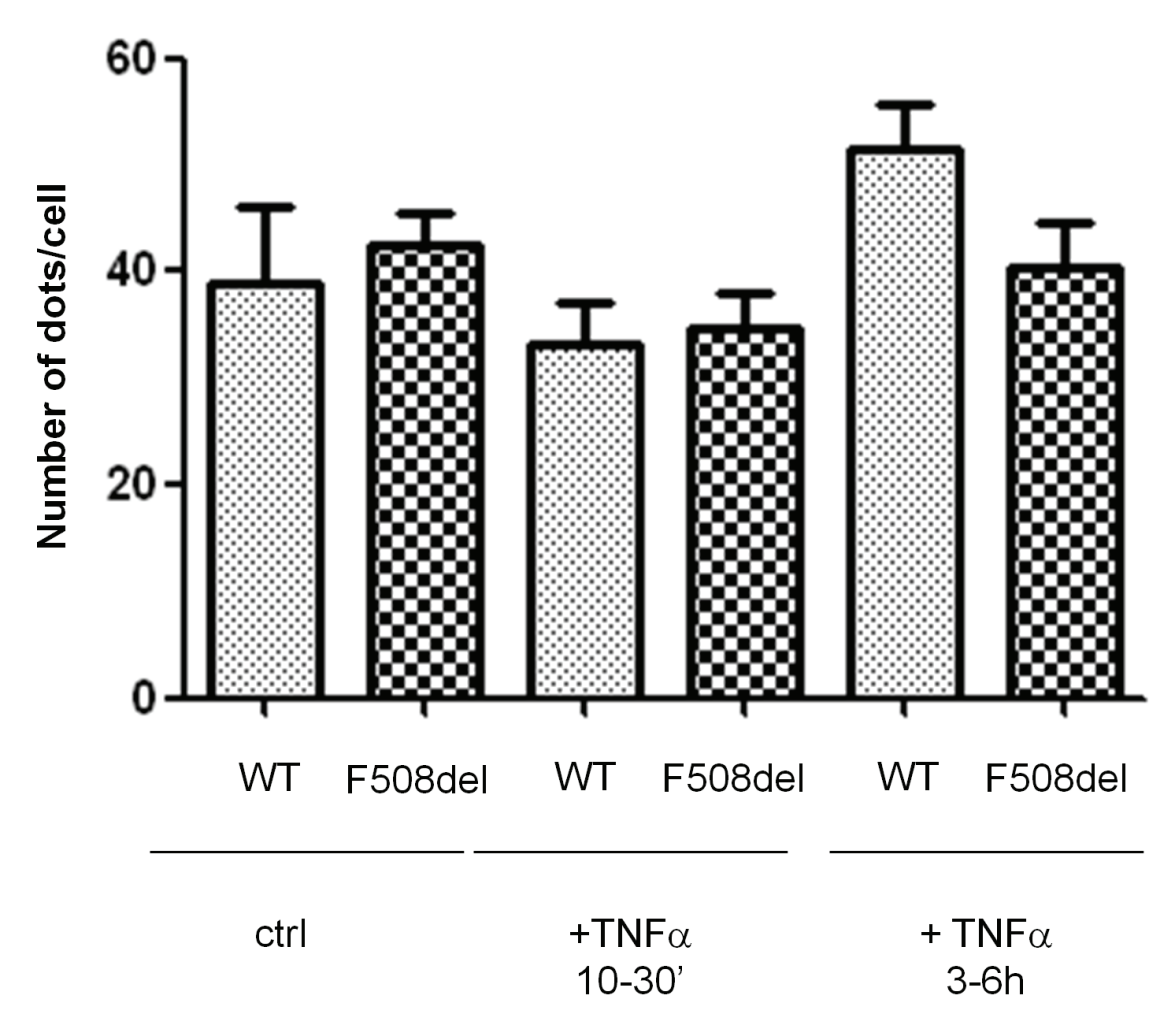
Effect of TNFα on F508del-CFTR-NHERF1 proximity in expressing F508del-CFTR HeLa cells. Number of pairs F508delCFTR-NHEFR1 in controls in TNFα-treated (as indicated) cells; Number of cells for control NHERF1-WTCFTR: 39 ± 13 n=90, NHERF1-F508del-CFTR: 42 ± 6, n= 80 cells; cells treated for 30min: NHERF1-WTCFTR 33 ± 7.7 n= 114 cells, NHERF1-F508del-CFTR 35 ± 7.1 n= 106; treated for 3 h: NHERF1-WTCFTR: 51 ± 9.1 n= 80 cells, NHERF1-F508del-CFTR: 40 ± 8.2 n=104 cells, p= NS in all conditions.

As rescued F508del-CFTR still carries a misfolding mutation, it will be recognized by the peripheral quality control ^19^. Therefore, we wanted to evaluate the effect of TNFα on the stability of rescued F508del-CFTR. Because it was shown that the adaptor protein NHERF1 stabilizes CFTR at the plasma membrane ^20^, we investigated whether the number of NHERF1–F508del-CFTR protein pairs was modified by TNFα treatment. Using the PLA assay, we observed that the former did not significantly change under TNFα treatment. These results indicate that, while TNFα enables the exit of F508del-CFTR from the ER, it does not alter the peripheral quality control ( Supplementary Figure S1).

Raw data for Bitam et al., 2015 ‘An unexpected effect of TNFα on F508del-CFTR maturation and function.’ Raw dataset 1:                   HeLa cells stably transfected with the plasmid F508del-CFTR were used in this experiment. a) The first lane represents F508del-CFTR HeLa cells non-treated. The second lane represents F508del-CFTR HeLa cells treated with TNFa at 50ng/ml for 10 min. The third lane represents F508del-CFTR HeLa cells treated with TNFa at 50ng/ml for 3h.The fourth lane represents F508del-CFTR HeLa cells treated with TNFa at 50ng/ml for 24h. The fifth lane is not relevant for this experiment. The last lane represents HeLa cells non transfected with markers of weight. The anti-CFTR used is MM-13-4 (mouse antibody). b) The membrane has been stripped and the a-tubulin has been used. This is represented by the second western blot. Stripping procedure: after the first detection of CFTR proteins on the blot, the nitrocellulose membrane is incubated for 30 min in a stripping buffer containing 2% SDS, 625mM TRIS pH 6.7, then the membrane is washed 3 times with PBS during 10 minutes. Next, the membrane is blocked again as described in the protocol of western blot, followed by the use of new first antibody and detected as described in the protocol of western blot.           
Raw dataset 2:                        First sheet: Raw data for Figure 1B
HeLa cells stably transfected with the plasmid F508del-CFTR were used in this experiment.
·            The first table (in orange) represents F508del-CFTR HeLa cells non-treated.
The lane A represents the number of the experiment, for the table orange: 8 experiments have been done. The intensity of band C and band B have been quantified with ImageJ software (see methods for version). The intensities measured are shown in the column C and D. The column E represents the ratio: intensity of the band C/ (intensity of band B+ intensity of band C).
The square G5 represents the mean of C/C+B.
The square G6 represents the SD of the mean.
·            The second table (yellow) presents the individual values obtained in F508del-CFTR HeLa cells treated with TNFa at 50ng/ml for 10’.
·            The third table (bleu) presents the individual values obtained F508del-CFTR HeLa cells treated with TNFa at 50ng/ml for 3h.
·            The forth table (pink) represents the individual values obtained F508del-CFTR HeLa cells treated with TNFa at 50ng/ml for 24h.
Second sheet: Raw data for Figure 1D
·            HeLa cells stably transfected with the plasmid F508del-CFTR were used in this experiment.
·            The first table (yellow) represents F508del-CFTR HeLa cells non-treated.
·            The column A represents the number of the experiment: 4 experiments have been done. The intensity of band C and of band B have been quantified with ImageJ software v1.47. The results are in the column B and C. The column D represents the ratio: intensity of the band C/ (intensity of band B+ intensity of band C).
·            The square F12 represents the mean of C/C+B.
·            The square F13 represents the SD of the mean.
Third sheet: Raw data for Figure 8B
·            HeLa cells stably transfected with the plasmid F508del-CFTR were used in this experiment.
·             The column C represents the conditions of the experiments
·             The blue table represents F508del-CFTR HeLa cells non-treated.
·            The pink table represents F508del-CFTR HeLa cells treated with 50ng/ml of TNFa during 30 min.
·            The yellow table represents F508del-CFTR HeLa cells treated with 50ng/ml of TNFa during 3h.
·            Column D represents the number of dots counted in the area chosen and the column E the number of cells counted in the area.
·            Blue square:
·            F7-F9: is the number of dots counted divided by the number of cells.
·            Orange square is the average of the means.
·            Green square is the SD of the means.
Fourth sheet: Raw data for Supplementary figure S1
HeLa cells stably transfected with the plasmid WT-CFTR and F508del-CFTR were used in this experiment. Different conditions have been tested: 50ng/ml of TNFa at different times: 30’-3h.
Blue represents controls: non-treated cells either: WT or F508del-CFTR HeLa cells.
Pink represents cells treated with 50ng/ml of TNFa for 30’.
Green represents cells treated with 50ng/ml of TNFa for 3h.
Column C: C8-10, C16-19, C24-28 represents the number of cells counted in the area for WT-heLa cells
Column I: I8-11, I16-20, I26-30 represents the number of cells counted in the area for F508del-CFTR.
Column D: D8-10, D16-19, D24-28 represents the number of cells counted in the area for WT-HeLa cells.
Column J: J8-11, J16-20, J26-30 represents the number of cells counted in the area for F508del-CFTR HeLa cells.
Column E: E8-10, E16-19, E24-28 represents the number of cells counted in the area treated with 50ng/ml of TNFa for 3h.
Column K: K8-11, K16-20, K26-30 represents the number of cells counted in the area for F508del-CFTR HeLa cells.
Totals of dots per area are divided by total of dots per area for each condition.
Means and SD are calculated and sum up in the last table.
SD are in purple and means in orange.
Raw dataset 3:            Table “BasaI I_CFTR_/C”. The normalized with regard to cell surface evaluated by measuring the cell capacity (C, pF) values of I_CFTR_ current values obtained in control, non-treated conditions, used to calculates the mean values showed in Fig 3B (between -100mV and +80mV) the mean values for each imposed voltage are presented in the bottom of the corresponding column. The values at -60mV are in blue as they served for the statistics shown in Figure 3C
Table “10 min 50 ng/ml TNFa I_CFTR_/C”. See legend for Table “BasaI I_CFTR_/C”. Here the cells were exposed to 50 ng/ml TNFa for 10 min.
Raw dataset 4:            The normalized current values measured in individual cells which were used to calculate the mean currents shown in Figure 3C. Each cell served as its own control. The currents were measured after 10 min of perfusion with cAMP cocktail (CPTAMPc and IBMX, see methods), then the TNFa was added to the perfusion in the presence of cAMP cocktail for next 10 min followed by perfusion of CFTR inh 172 (see methods and legends of fig 3C for details).
Raw dataset 5:         Individual values of normalized I_CFTR_/C for dose-response of 0 to 50 ng/ml TNFα after 10 min: CFTR current amplitudes were recorded at -60 mV and normalized to cell capacitance.
Raw dataset 6:        Table “I _CFTR_ /C (pA/pF) with IL1b + cAMP/IBMX”.  Shown are the values of I_CFTR_ current, normalized with regard to the cell surface evaluated by measuring the cell capacity (C, pF) in cells treated with IL1b in the perfusion for 10 min after activation of the baseline current with a cocktail of CPTcAMP/IBMX. These values were used to calculate the mean values shown in the I/V curve in Figure 4 (between -100 mV and +80 mV). The mean values and the standard error of the mean for each imposed voltage are presented in the bottom of the corresponding column. The values at -60 mV are shown in blue as they served for the histogram shown in Figure 4 (right panel). Table “I _CFTR_ /C (pA/pF) cAMP/IBMX control”. See legend for table “I _CFTR_ /C (pA/pF) with IL1b + cAMP/IBMX”. The cells were exposed only to CPTcAMP/IBMX without IL1b and served as controls. Table “I _CFTR_ /C (pA/pF) at -60 mV”. Depicted are the individual normalized current values at -60 mV measured in individual cells shown in table “I _CFTR_ /C (pA/pF) with IL1b + cAMP/IBMX” and “I _CFTR_ /C (pA/pF) cAMP/IBMX control”, respectively, which were used to calculate the mean currents shown in the histogram of Figure 4 (right panel). A different set of cells served for control and IL1b treated cells. The values for TNFa treated cells are the same as shown in Fig 3C and in the raw data for Figure 3.
Raw dataset 7:         As for the legend for Figure 3B raw data except that the cells were pretreated with BFA (see methods and text for details). The column -60mV is in blue as these values served for histograms shown in Fig 6C.
Raw dataset 8:         Individual values of normalized I_CFTR_/C for cells pre-treated or not with BFA (see methods and text for details). After pre-treatment with BFA the cells were exposed to TNFa for 10 min: CFTR current amplitudes were recorded at -60 mV and normalized to cell capacitance.
Raw dataset 9:         See legends “Fig 3B raw data” except that the cells were pretreated with GF109203X for 30min (see methods and text for details). The column -60mV is in blue as these values served for histograms shown in Fig 8C.
Raw dataset 10:       Individual values of normalized I_CFTR_/C for cells pre-treated or not with GF109203X (see methods and text for details). After pre-treatment with GF109203X the cells were exposed to TNFa for 10 min: CFTR current amplitudes were recorded at -60 mV and normalized to cell capacitance. Click here for additional data file. Copyright: © 2015 Bitam S et al. 2015 Data associated with the article are available under the terms of the Creative Commons Zero "No rights reserved" data waiver (CC0 1.0 Public domain dedication).

## Discussion

In this study we demonstrate a novel, rapid and unexpected effect of TNFα on F508del-CFTR trafficking, maturation and function as a chloride channel. This effect was observed in HeLa cells stably transfected with F508del-CFTR, and in HBE cells derived from homozygous F508del CF patients in primary culture, giving credence to the physiological relevance of this effect. Our data suggest that TNFα – induced I _CFTR_ is due to the release of misfolded F508del-CFTR from the ER to the Golgi apparatus and the subsequent insertion of late Golgi vesicles into the plasma membrane. This TNFα action was found to be dependent on PKC activity. In HeLa cells expressing F508del-CFTR, the TNFα – induced I _CFTR_ activity is transient, but in CF patients’ cells it lasted for 24h, suggesting that it may occur during chronic inflammation.

TNFα has been extensively described to play a major role in the inflammatory process by inducing cytokine release from inflammatory cells as well as bronchial epithelial cells ^21, 22^. TNFα induces IL-8 and IL-1β synthesis and secretion from adenocarcinoma lung cancer cells, Calu-3 cells ^1^. IL-1β has recently been reported to stimulate the expression of CFTR in T84 colon carcinoma cells, through NF-κB signalling ^23^. For these reasons, possible involvement of other inflammatory mediators in the F508del-CFTR response to TNFα could not be excluded. However, using a similar protocol as for TNFα, we report here that IL-1β, another pro-inflammatory cytokine, did not affect I _CFTR_. Therefore, the effect of TNFα on F508del-CFTR, described here, seems to be specific to this cytokine, and is most likely distinctive of its stimulatory effect on pro-inflammatory cytokines.

The role of TNFα in enhancing chloride transport through F508del-CFTR is consistent with its function in immunity. Indeed, host defense and efficient mucociliary clearance is achieved by the stimulation of chloride transport and subsequent regulation of airway surface hydration. Other mediators have been reported to play a role in epithelial transport, including pro-inflammatory mediators, such as prostaglandins, leukotrienes and interferon gamma ^24– 26^ as well as pro-resolution mediators ^27^. In other models, such as the colon adenocarcinoma-derived cell line T84, the same treatment reduced CFTR expression and function ^6^. In the present study, the TNFα-induced F508del-CFTR activity was transient in HeLa cells, but in HBE cells from CF patients this effect was sustained over 24h. Taken together our data provide evidence for a novel effect of TNFα in stimulating F508del-CFTR maturation and activation during both the acute and chronic phases of inflammation.

The rapid insertion of membrane proteins into the plasma membrane following short-term treatment by TNFα has been previously described for other membrane proteins. For example, it was observed for the leptin receptor, a primary regulator of leptin signaling believed to regulate energy homeostasis, reproduction and immunity ^10^, and for an injury-promoting receptor in motor neurons, the α-amino-3-hydroxy-5-methyl-4-isoxazolepropionic acid (AMPA) type glutamate receptor, involved in amyotrophic lateral sclerosis ^28^. Both proteins are inserted into plasma membrane in a PKC-dependent manner, by a mechanism that may be common, at least in part, to the one uncovered by our study on F508del-CFTR. However, there is a marked difference between these studies and our observations. In the case of leptin receptor and AMPA, it is the correctly-folded proteins that are inserted into plasma membrane in response to TNFα treatments. On the contrary, TNFα has no effect on WT-CFTR, whereas it promotes insertion of an abnormally folded and prematurely degraded protein, F508del-CFTR. Therefore, our results suggest that the underlying mechanisms of action between the effect of TNFα on wild-type proteins and abnormally folded proteins must differ. One possible explanation is a differential regulation of kinases. Our preliminary results indicate that ERK2 phosphorylation is diminished by TNFα within 10min and remains low for 24h. It is therefore possible that the PKC pathway, involved in F508del-CFTR translocation to the plasma membrane leads to the decreased phosphorylation of ERK2. Indeed, the fact that one of PKC isoforms, PKCδ, activates ERK supports this hypothesis ^29^. Nevertheless, the phosphorylation status of ERK1/2 in the context of transepithelial ion transport in the presence of TNFα has not been investigated. Conversely, other authors ^8^ have reported that ERK1/2 are not involved in the TNFα-induced decrease in transepithelial resistance of human epithelial cells, and in the prevention of these effects by probiotics, although they did not determine the phosphorylation status of the kinases ^8^. In any case, these observations would concern WT-CFTR, i.e. the properly folded protein, which under our experimental conditions is not regulated by TNFα. Of note, the chronic treatment of intestinal cells by TNFα (>24h) leads to decreased expression of WT-CFTR ^6, 30^. Thus, our study opens a new field of investigation into those signaling pathways activated by TNFα and/or other cytokines during the maturation of wild-type and misfolded proteins.

The translocation of F508del-CFTR to the plasma-membrane upon exposure to TNFα and the inhibitory effect of BFA on TNFα-activated I _CFTR_ suggest that TNFα-induced insertion of vesicles containing F508del-CFTR proteins from Golgi into plasma membrane enhances cAMP-dependent chloride currents (I _CFTR_). Conversely, the keratin 8–F508del-CFTR protein complex recently shown by us as an unwanted interaction preventing the escape of F508del-CFTR from the degradation pathways ^17, 31, 32^ seems not to be involved in this process.

TNFα acts on the trafficking of F508del-CFTR through the Golgi apparatus since blocking of vesicular exit from ER by BFA prevents the development of I _CFTR_ ( Figure 6). At later times, F508del-CFTR may be stabilized at the plasma membrane by favoring the formation of a protein macrocomplex through interaction with NHERF1. This is supported by two observations: first, it has previously been reported that a multiprotein complex (NHERF1-CFTR-ezrin-actin) plays a significant role in maintaining tight junction organization and function in cystic fibrosis epithelial cells ^33^. Second, it is known that NHERF1 itself prevents F508del-CFTR from degradation ^20^. Even if the number of F508del-CFTR-NHERF1 protein complexes was not changed by short term treatment with TNFα, NHERF1 and/or other proteins that bind to the PDZ domain of CFTR might play a stabilizing role. Within this line of investigation, we and others have demonstrated that CFTR forms a protein complex with TNFα receptor, p11, Annexin 1 and cPLA2 ^1, 34^. Within 10 min TNFα treatment is sufficient to relocate CFTR, together with these four proteins, to lipid raft-like detergent-resistant microdomains. How this mechanism relates to the observations described in the present study and to the potential implication of NHERF1 remains to be investigated.

In agreement with our observations, all studies related to the rapid effects of TNFα on membrane proteins mentioned in this manuscript ^1, 10, 28, 34^ suggest that this pro-inflammatory cytokine very rapidly modifies the composition of the plasma membrane, which may (in the case of F508del-CFTR) lead to profound changes in ion transport. It has also been reported that VX-809, a corrector for F508del-CFTR, stabilizes NHERF1-F508del-CFTR at the plasma membrane ^35^. We propose that TNFα is one of the players in the stabilization of F508del-CFTR at the plasma membrane, at least for 24h after the onset of inflammation. It is tempting to hypothesize that either TNFα behaves as VX-809 or TNFα and VX-809 actions could act in parallel.

Our observations are important as they highlight a novel perspective on airway inflammation in the context of CF that could open unexpected avenues in the understanding of correcting mechanisms. Indeed, it signifies that TNFα action is, at least, not opposed to the treatment. It has to be remembered, however, that during chronic inflammation, other mediators may have different behaviors. We propose that systematic studies on acute and chronic effects of inflammation mediators on F508del-CFTR trafficking and I _CFTR_ should be undertaken in the context of correcting treatments.

Finally, the effect of TNFα on F508del-CFTR maturation may provide a partial explanation for the residual activity of F508del-CFTR in patients with a mild CF phenotype ^9^. We propose systematic testing in CF patients of TNFα levels and, when possible, association of these tests with nasal potential measurements, systematic immunocytochemistry of F508del-CFTR in nasal cells, and determination of TNFα blood concentration. A potential correlation between these parameters and CF phenotype could be useful as a prognostic marker of disease evolution.

In summary, a corrector-like effect of TNFα on F508del-CFTR raises the question of the role played by acute inflammation in CF patients during treatment with correcting compounds.

## Data availability

The data referenced by this article are under copyright with the following copyright statement: Copyright: © 2015 Bitam S et al.

Data associated with the article are available under the terms of the Creative Commons Zero "No rights reserved" data waiver (CC0 1.0 Public domain dedication). 

*Figshare:* Raw data for Bitam *et al.*, 2015 ‘An unexpected effect of TNFα on F508del-CFTR maturation and function.’. doi: 10.6084/m9.figshare.1476156 ^36^
