# Supplementary figures and images for: An unexpected effect of TNF-α on F508del-CFTR maturation and function
Source: F1000Res. 2015 Sep 2;4:218. Originally published 2015 Jul 10. [Version 2] doi: 10.12688/f1000research.6683.2 (PMC4648213; doi:10.12688/f1000research.6683.2)

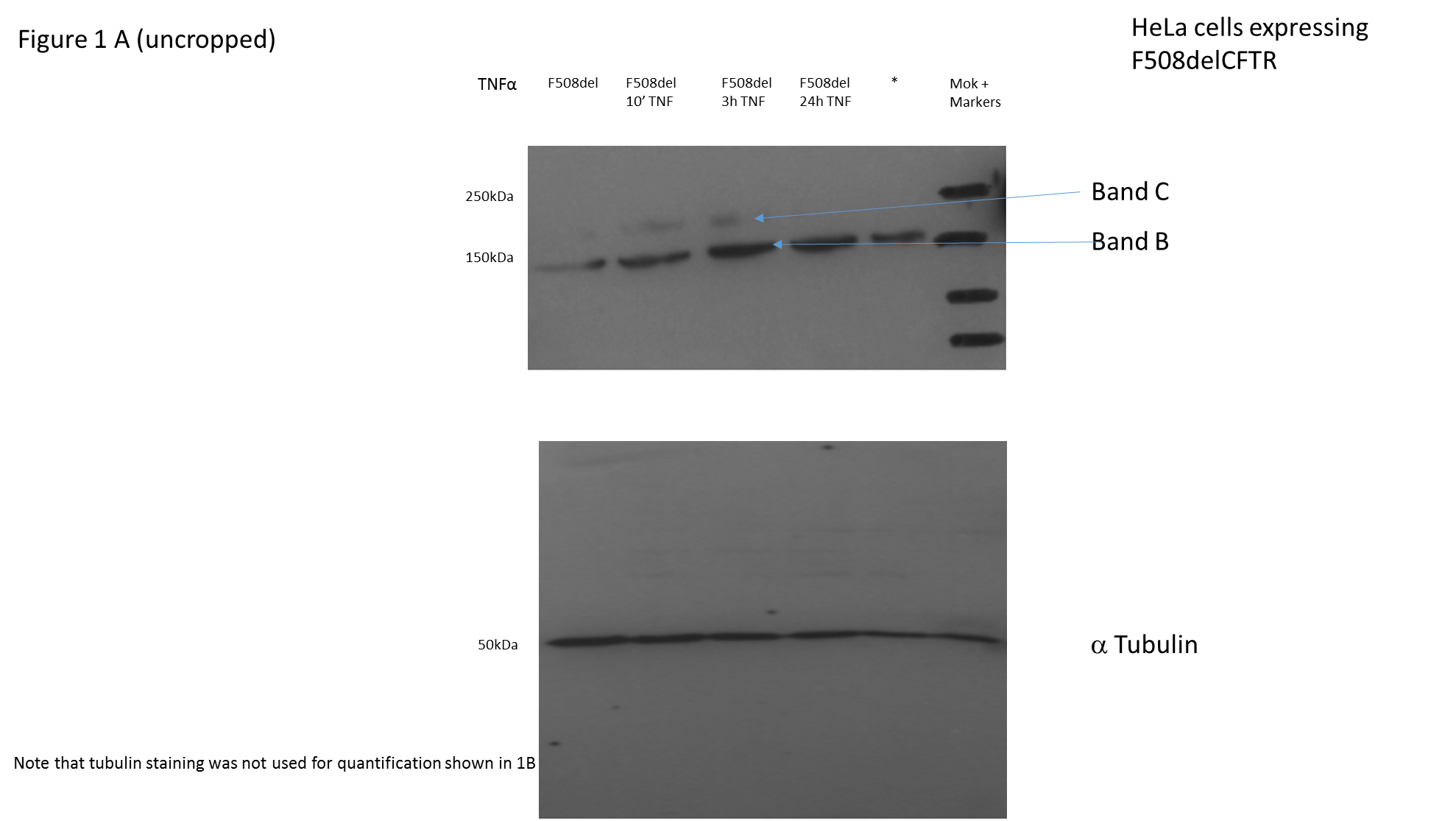

Supplement: Raw data for Bitam et al., 2015 ‘An unexpected effect of TNFα on F508del-CFTR maturation and function.’ — Raw dataset 1: HeLa cells stably transfected with the plasmid F508del-CFTR were used in this experiment. a) The first lane represents F508del-CFTR HeLa cells non-treated. The second lane represents F508del-CFTR HeLa cells treated with TNFa at 50ng/ml for 10 min. The third lane represents F508del-CFTR HeLa cells treated with TNFa at 50ng/ml for 3h.The fourth lane represents F508del-CFTR HeLa cells treated with TNFa at 50ng/ml for 24h. The fifth lane is not relevant for this experiment. The last lane represents HeLa cells non transfected with markers of weight. The anti-CFTR used is MM-13-4 (mouse antibody). b) The membrane has been stripped and the a-tubulin has been used. This is represented by the second western blot. Stripping procedure: after the first detection of CFTR proteins on the blot, the nitrocellulose membrane is incubated for 30 min in a stripping buffer containing 2% SDS, 625mM TRIS pH 6.7, then the membrane is washed 3 times with PBS during 10 minutes. Next, the membrane is blocked again as described in the protocol of western blot, followed by the use of new first antibody and detected as described in the protocol of western blot. Raw dataset 2: First sheet: Raw data for Figure 1B HeLa cells stably transfected with the plasmid F508del-CFTR were used in this experiment. · The first table (in orange) represents F508del-CFTR HeLa cells non-treated. The lane A represents the number of the experiment, for the table orange: 8 experiments have been done. The intensity of band C and band B have been quantified with ImageJ software (see methods for version). The intensities measured are shown in the column C and D. The column E represents the ratio: intensity of the band C/ (intensity of band B+ intensity of band C). The square G5 represents the mean of C/C+B. The square G6 represents the SD of the mean. · The second table (yellow) presents the individual values obtained in F508del-CFTR HeLa cells treated with TNFa at 50ng/ml for 10’. · The third table (bleu) [file f1000research-4-7555-s0000.tgz › Uncropped_gel_images_for_Figures_1a_and_b.tif]
