# Supplementary material for: An unexpected effect of TNF-α on F508del-CFTR maturation and function
Source: F1000Res. 2015 Sep 2;4:218. Originally published 2015 Jul 10. [Version 2] doi: 10.12688/f1000research.6683.2 (PMC4648213; doi:10.12688/f1000research.6683.2)
Supplement: Peer review history

# Peer review: Referee response for version 1

**DOI:** 10.5256/f1000research.7179.r9637

**Published:** 2015-08-25

**Authors:** Bruno Miroux

**Affiliations:** 1 Laboratory of Physical and Chemical Biology of Membrane Proteins, Paris Diderot University, Paris, France

In this report, Bitam *et al. *investigated the putative correcting function of TNFα in the targeting of DF508-CFTR protein to the plasma membrane. Experiments were performed on two different biological model systems: Hela stable cell lines expressing either wt or DF508-CFTR protein and a more physiologically relevant system, i.e. primary Human Bronchial Epithelial cells (HBE), isolated from bronchial explants of CF and non-CF patients after lung transplantation. In the first part of the study, they not only convincingly showed that TNFα increases cell surface expression of F508del-CFTR in HeLa cells but also that it restores the chloride conductance function of CFTR. To prove the physiological relevance of these initial findings the authors performed the same acute treatment on HBE cells and showed that it increases the expression of F508del-CFTR at the plasma membrane in a functional state. More importantly, in this model, acute stimulation of cells with TNFα had a long lasting effect, up to 24H, suggesting that TNFα could be useful for the treatment of the disease. The second part of the paper is devoted to the search of TNFα mechanism of action. Using inhibitors of various signaling pathways they showed that TNFα acts at least at two different levels: vesicular trafficking and PKC dependent signaling pathway. Altogether these set of data revealed a very interesting and intriguing correcting function of TNFα in the targeting of F508del-CFTR to the plasma membrane. Experimental evidences are statistically robust and well described. The paper is overall well written and discussed and deserves indexation.

I have read this submission. I believe that I have an appropriate level of expertise to confirm that it is of an acceptable scientific standard.

# Peer review: Referee response for version 1

**DOI:** 10.5256/f1000research.7179.r9762

**Published:** 2015-08-03

**Authors:** Renaud Beauwens

**Affiliations:** 1 Laboratory of Cellular and Molecular Physiology, Free University of Brussels, Brussels, Belgium

Cystic Fibrosis is one of the most common genetic diseases attributable to mutations in the cystic fibrosis transmembrane regulator ( *cftr*) gene which encodes a cAMP-dependent Cl ^-^ channel, the CFTR protein present in the apical membrane of numerous epithelia. Inflammation is a hallmark of the disease contributing to its severity and has been suggested to arise independently of bacterial infection. The study by Bitam *et al*. questions whether mediators of inflammation affect CFTR expression and ion transport function. The most frequent mutation DF508 leads to a functional protein that is prematurely degraded. The authors investigated the effect of tumor necrosis factor a (TNFa), a proinflammatory cytokine in DF508-CFTR-transfected HeLa cells as well as in human bronchial cells expressing DF508-CFTR in primary culture. In both cases, exposure to TNFa (0.5 – 50 ng/ml) for 10 min enhanced the maturation of DF508-CFTR with appearance of its glycosylated form at the plasma membrane and CFTR-mediated chloride current inhibitable by the specific CFTR inhibitor (CFTR _inh_ 172). The effect was already observed within 10 min and lasted at least 24 h. It involves binding to TNFa receptor, activation of protein kinase C and vesicular trafficking to the plasma membrane.  TNFa induced no change in expression of WT CFTR in control cells.

In summary this study shows that TNFa behaves as a corrector molecule. If sustained treatment also lead to similar increased maturation at low dose of TNFa, it could even be considered as a possible additive in the therapeutic armamentarium.

The study is very interesting, well done and well-illustrated by confocal microscopy and it certainly deserves indexing.

I have read this submission. I believe that I have an appropriate level of expertise to confirm that it is of an acceptable scientific standard.
